# Phytochemical Profiling and Bio-Potentiality of Genus *Scutellaria*: Biomedical Approach

**DOI:** 10.3390/biom12070936

**Published:** 2022-07-04

**Authors:** Muddaser Shah, Sidra Mubin, Syed Shams ul Hassan, Priti Tagde, Obaid Ullah, Md. Habibur Rahman, Ahmed Al-Harrasi, Najeeb Ur Rehman, Waheed Murad

**Affiliations:** 1 Department of Botany, Abdul Wali Khan University Mardan, Mardan 23200, Pakistan; muddasershah@awkum.edu.pk; 2 Natural and Medical Sciences Research Center, University of Nizwa, P.O. Box 33, Birkat Al Mauz, Nizwa 616, Oman; 3 Department of Botany, Hazara University Mansehra, Mansehra 21310, Pakistan; shahhu123@gmail.com; 4 Department of Natural Product Chemistry, School of Pharmacy, Shanghai Jiao Tong University, Shanghai 200240, China; shams1327@yahoo.com; 5 Shanghai Key Laboratory for Molecular Engineering of Chiral Drugs, School of Pharmacy, Shanghai Jiao Tong University, Shanghai 200240, China; 6 Amity Institute of Pharmacy, Amity University, Noida 201301, India; tagde_priti@rediffmail.com; 7 Department of Chemistry, University of Malakand, Chakdara 18800, Pakistan; obaidullah@unizwa.edu.om; 8 Department of Global Medical Science, Wonju College of Medicine, Yonsei University, Wonju 26426, Korea; pharmacisthabib@gmail.com

**Keywords:** natural products, antimicrobial, antioxidant, anticancer: anti-inflammatory, enzyme inhibitory activity, analgesic activity

## Abstract

*Scutellaria* (Lamiaceae) comprises over 360 species. Based on its morphological structure of calyx, also known as Skullcap, it is herbaceous by habit and cosmopolitan by habitat. The species of *Scutellaria* are widely used in local communities as a natural remedy. The genus contributed over three hundred bioactive compounds mainly represented by flavonoids and phenols, chemical ingredients which serve as potential candidates for the therapy of various biological activities. Thus, the current review is an attempt to highlight the biological significance and its correlation to various isolated bioactive ingredients including flavonoids, terpenoids, phenols, alkaloids, and steroids. However, flavonoids were the dominant group observed. The findings of the *Scutellaria* reveal that due to its affluent basis of numerous chemical ingredients it has a diverse range of pharmacological potentials, such as antimicrobial, antioxidant, antifeedant, enzyme inhibition, anti-inflammatory, and analgesic significance. Currently, various bioactive ingredients have been investigated for various biological activities from the genus *Scutellaria* in vitro and in vivo. Furthermore, these data help us to highlight its biomedical application and to isolate the responsible compounds to produce innovative medications as an alternative to synthetic drugs.

## 1. Introduction

The genus *Scutellaria* is comprised of over three hundred and fifty (350) plant species, cosmopolitan by habitat, and its species have served as a natural remedy since ancient times throughout the globe [1]. Therapeutic herbs have been proven to be a significant origin of medication for the mitigation of human diseases [2]. According to the survey report by the World health organization (WHO), approximately 80% of the population of the world is dependent on herbal-based conventional remedies for primary health care [3]. Herbal medicines have grown in popularity and acceptance in the Western world annually. Many people want a better lifestyle, placing their trust in ayurvedic and alternate therapies as natural, reliable, and efficient. Americans spent more than USD 12 billion on natural additions and more than USD 27 billion on natural products in 1998 [4]. For instance, 125 million of 500 million prescriptions by physicians each year involve preparation derived from natural sources.

Some species of the genus *Scutellaria* have been extensively used as essential remedies in conventional treatment in countries such as North America, China, Korea, and most European countries [5]. New studies have also validated the plant’s conventional applications [6]. *Scutellaria* appears to have over 360 species globally, with 20 species and two hybrids increasing in Iran. Ten native species and two hybrids comprise the species [7]. The species of *Scutellaria*, *Scutellaria pinnatifida*, have five subspecies. One among *Scutellaria pinnatifida* is endemic to Iran. The involvement of some *Scutellaria* species in Iran prompted us to investigate the cytotoxic and apoptotic stimulation of *Scutellaria pinnatifida* on human leukemia cancer cells [8]. In clinical trials, *Scutellaria* has been considered as a treatment or additional relief in handling several breast and prostate cancers with minimal toxicity in the various dosage form. In the genus *Scutellaria*, many plant species were reported with flavonoids as a major group of chemical ingredients [9], which can cure cancer [10]. *Scutellaria baicalensis* is a well-known traditional Chinese medicine whose chemical and pharmacological properties have been extensively researched. *Scutellaria baicalensis* has antibacterial, anticancer, antiviral, and antiallergic properties [11]. The recent findings depicted that *Scutellaria* species have diverse biomedical practices. *Scutellaria* species are locally known to treat various complications as they contain many chemical ingredients, such as baicalin, wagonins, and scutellarin, which have a key role in the production of drugs [9]. Thus, a well-developed procedure is considered necessary to isolate the active constituents in large amounts without affecting their quality [12,13]. However, additional exploration at the molecular and cellular levels is needed to develop an alternative approach in order to meet the local and international demands as plant species decrease with time using artificial cultivation [14,15]. The plant species of the genus *Scutellaria* are widely distributed in north China, Japan, Russia, and Pakistan. In Pakistan, the *Scutellaria* species are mainly found in temperate regions of Districts Swat, Chitral, Mansehra, and Parachinar on the tail of mountains and prefer to grow in moist loamy soils. Locally the plant species are used for their numerous properties, such as antimicrobial, antioxidant, anti-inflammatory, and analgesic, and the leaves of some plants of the mentioned genus are used to make green tea (kava) [10]. Some of its species are used to cure diabetes [16]. Some of the species of the genus *Scutellaria* such as *S. baicalensis* are native to the Korean peninsula, China, Japan, Mongolia, and Russia. Due to higher demand in recent years, it is now grown globally in China’s Shandong, Hebei, Shanxi, Inner Mongolia, and Gansu provinces. It should be mentioned that an allied species’ herb, *Scutellaria barbata*, has been used in the Chinese medicine Ban-Zhi-Lian [17]. Şenol et al. investigated 33 *Scutellaria* species from Turkey and found that the level of these physiologically active compounds in dry extracts of *Scutellaria galericulata* and *Scutellaria albida* had a similar relationship [18]. Park et al. revealed the influence of various carbohydrate sources on the type and number of flavonoids accumulated in hairy root cultures of *Scutellaria baicalensis* [19]. Long-term administration of 2500 mg/kg of *Scutellaria baicalensis* increased liver injury in female rats [20]. Therefore, it is notable that after prolonged use of *Scutellaria baicalensis,* blood glucose levels may increase even after ceasing to take the drug [20]. Şenol et al. investigated 33 *Scutellaria* species from Turkey and found that the level of these physiologically active compounds in dry extracts of *Scutellaria galericulata* and *Scutellaria albida* had a similar relationship [18]. Park et al. revealed the influence of various carbohydrate sources on the type and number of flavonoids accumulated in hairy root cultures of *Scutellaria baicalensis* [19]. Long-term administration of 2500 mg/kg of *Scutellaria baicalensis* increased liver injury in female rats [20]. Therefore, it is notable that after prolonged use of *Scutellaria baicalensis* blood glucose levels may increase even after ceasing to take the drug [20].

**Figure 1:**
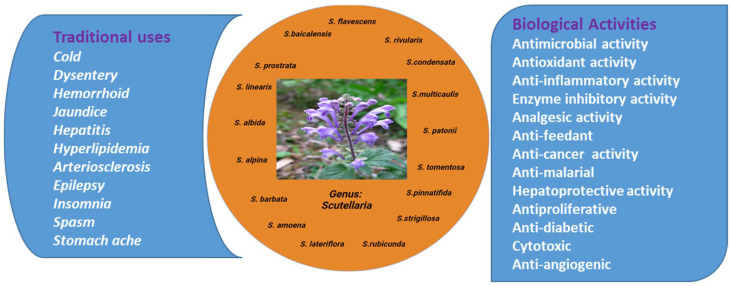
Traditional and biological applications of the genus *Scutellaria*.

Various plant species of the genus *Scutellaria* serve as a local remedy to treat anxiety, cancer, jaundice, cirrhosis, hepatitis, and neurological problems. PubMed, Springer, Elsevier, and Chinese herbal classics were searched for peer-reviewed works published in the last few years. The emphasis of this study is on the pharmacologically active chemicals isolated from *Scutellaria* and studied in vitro and in vivo. This review article comprises a compilation of research publications and reviews articles. Due to its high well-documented phytochemical significance, *Scutellaria* is an excellent candidate for the expansion of remedial model species. *Scutellaria* species are thus exceptional platform schemes for learning the biochemistry of medicinal plants. The significance of the *Scutellaria* and its therapeutic uses evaluated through in vitro and in vivo analysis is given in detail) in this article [17]. Thus, the current review highlights the biological significance of *Scutellaria* and its correlation to various isolated bioactive ingredients including flavonoids, terpenoids, phenols, alkaloids, and steroids. The traditional and biological activities of the genus *Scutellaria* is illustrated in Figure 1. 

## 2. Compounds with Biological Activities from Genus *Scutellaria* Species

The genus *Scutellaria* is an affluent source of bioactive ingredients representing alkaloids, flavonoids, terpenoids, phenols, and steroids. Flavonoids are the dominant group, followed by the terpenoids. Furthermore, some of the compounds identified from the genus *Scutellaria* are given in Table 1. 

## 3. Most Prominent Compounds of Genus *Scutellaria*

**Figure 2:**
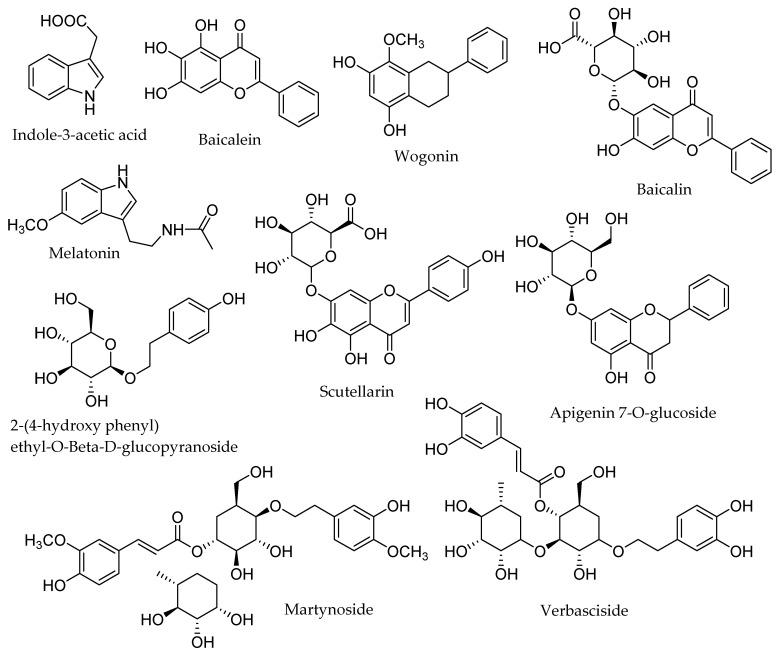
Dominant compounds isolated from *Scutellaria* species.

The most prominent compounds isolated from *Scutellaria* species are baicalin, baicalein, and wogonin. Figure 2 reflects the dominant chemical ingredients contributed by the genus *Scutellaria*, which have wide pharmacological properties, including antioxidant, analgesic, laxative, diuretic, anti-diabetic, anti-allergy, anti-inflammatory, and regulator of lipid and arachidonate metabolism [1,76]. 

The chemical constituents of medicinal plants are mainly influenced by various environmental gradients. 

The study described by Malikov and Yuledashev [77] revealed that approximately 208 bioactive ingredients were isolated and identified as representing phenols from *Scutellaria* species and mainly contributed by *S. baicalensis*. In addition, Shah et al. [1] described the tentative identification of 16 compounds from *S. edelbergii*. Baicalin and baicalein are the major compounds reported from *Scutellaria*. Baicalin is a flavonoid with non-nucleoside reverse transcriptase inhibitor (NNRTI) action, containing various bioactive compounds. Platelet 12-lipoxygenase-1, lipid peroxidation, and cell growth in human hepatocellular carcinoma cell lines are all inhibited by baicalein [78]. Bioactive ingredients wogonin, scutellarin, and scutellarin are the main constituents isolated from the genus *Scutellaria* and depicted promising therapeutic significance [79]. Recent investigations have highlighted the promising therapeutic potential of wogonin and promoted its practices to enhance its usage and applications in conventional remedies [80]. Wogonin also has anti-inflammatory significance and is a powerful ordinary neuroprotective composite that prevents the inflammatory activity of microglia. Some findings also reflected that wogonin reduced neuronal death and inhibited inflammation in animal models [81]. Previously, Scutellarin played a key role in treating a variety of sleep disorders, cardiovascular diseases, migraines, depression, and memory impairment through practices in China that use scutellarin for vasculature improvement, blood vessel dilation, blood platelet count reduction, and blood viscosity reduction [82]. Furthermore, *S. baicalensis* has also been found to have high melatonin levels. Melatonin has effective antioxidant properties, is a master regulator of circadian rhythms in various organisms, and is a hormone involved in neurological disorders like migraines and depression [45].

**Figure 3:**
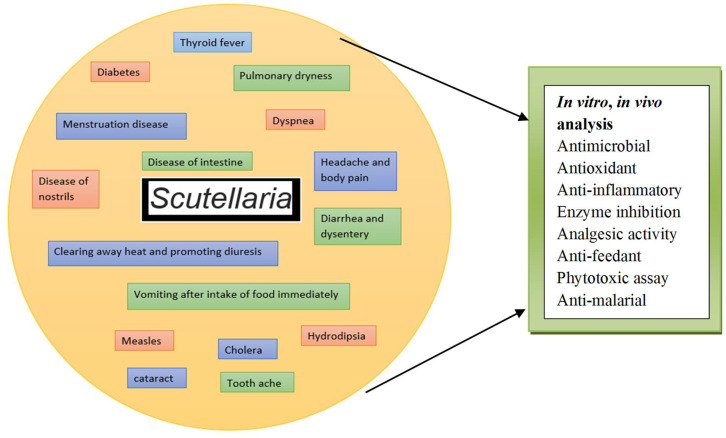
The traditional uses and biological activity of *S. baicalensis* in China.

Several Japanese Kampo remedy mitigation methods use *S. baicalensis* as an ingredient to treat disputes associated with circadian rhythm dysfunction, like seasonal moving syndromes and diabetic nocturnal polyuria [45]. Melatonin has been found in 108 different plant species used in traditional Chinese medicine. Some of the traditional uses of *S. baicalensis* in China are illustrated in Figure 3.

Melatonin, wogonin, baicalein, baicalin, and scutellarin’s medical efficacy highlights the importance of medicinal plants as a useful basis for the synthesis of newer anti-inflammatory, anti-cancer, and neuroprotective drugs [83]. 

## 4. Phytochemical and Biological Assessment of *Scutellaria*

Modern therapeutic studies on crude concentrated abstractions and isolated compounds of such a genus’ plants have verified a variety of biological activities, along with prolyl Oligopeptides anticonvulsant, hepatoprotective, inhibitory, and memory-enhancing effects [84]. Phytochemistry is a branch of biochemistry that deals with plants and plant products. Phytochemicals have a high antioxidant potential and are being studied for their potential health benefits. According to epidemiological and animal studies, eating fruits, vegetables, and whole grains regularly reduces the risk of a variety of diseases associated with oxidative damage. Natural antioxidants are classified into two types: in vitro and in vivo antioxidants. Polyphenols, flavonoids, isoflavonoids, cyanidins, phytoestrogen, terpenoids, carotenoids, limonoids, phytosterols, glucosinolates, and fibers are just a few examples of phytochemicals [85]. Phytochemicals from some *Scutellaria* species showed high cytotoxic activity on several human tumor cell lines using in vitro studies. *S. platystegia* aerial parts in methanolic presented significant potential to act as an antioxidant agent using 2, 2-diphenyl-1-pycryl hydrazyl (DPPH) assay [11]. The dichloromethane (DCM) fraction demonstrated antimalarial activity in a cell-free method, yielding a 50% inhibitory concentration [86].

Phytochemical analysis of methanol concentrated abstractions utilizing reverse phase HPLC and the nuclear magnetic resonance (NMR) instrument for isolation and characterization of pure compounds yielded 2-(4-hydroxyphenyl) ethyl-O-D-glucopyranoside as of 10% besides apigenin 7-O-glucoside, verbascoside, and martynoside as of 40% solid-phase extraction fraction. Verbascoside and martynoside were found to be common biochemical markers [87].

## 5. General Extraction Process

**Figure 4:**
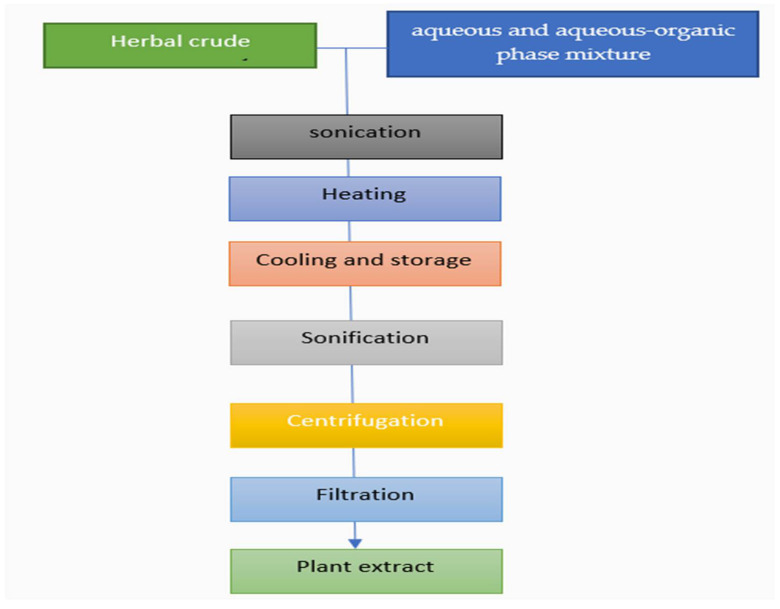
The schematic representation for the aqueous and aqueous-organic phase extraction preparation of the medical plants in general.

For crude extraction, water and 50% (*v*/*v*) ethanol in water were used. First, 2.0 g of ground plant material was mixed with 100 mL of distilled water (water extracts) or a 1:1 mixture of distilled water and ethyl alcohol (water-ethanolic extracts) containing 0.1 percent sodium hydrogen sulphate (water-ethanolic extracts). The resulting mixture was ultrasonically shaken for 15 min in an ultrasonic shaker before being heated at 80 degrees Celsius for 5 min. After bringing the mixture to room temperature, it was frozen at 4 degrees Celsius for 12 hrs. The mixtures were then resonicated and centrifuged for 10 min at 5000 rpm. Finally, the antioxidant and phenolic compound activity of the extracts can be determined. The schematic representation of the aqueous and aqueous-organic phase extraction preparation of the medicinal plants, in general, is given in Figure 4.

## 6. Biological Activities of Genus *Scutellaria*

### 6.1. Antimicrobial Capabilities

The antibiotic resistance by the microbes persuaded the researchers to devise a new alternative and effective antimicrobial agents which are much more effective with less adverse effects. The finding related to the essential oils of *Scutellaria* reveals the significant potential to act as an antimicrobial agent [88]. In addition to that, the essential oils of the *Scutellaria* contain bioactive ingredients such as eugenol, linalool, and other long-chain alcohols which can resist microbes. The finding of Shah et al. [5] reflected that the oils of the *Scutellaria edelbergii* have appreciable potential to act as an antimicrobial agent while the same plant various fractions offered appreciable antibacterial significance [10]. The data stated by Yu et al. [89] also provide us with information that the EOs of *S. barbata* has significant potential against the screened microbes *K. pneumoniae*, *S. maltophila*, *S. aureus*, *E. faecalis*, *S. marcescens*, *S. flexneri*, *C. freundii*, *S. paratyphi-A*, *S. simulans*, *S. heamolyticus*, *E. coli*, *P. aeruginosa*, *S. epidermidis*, *S. liquefaciens*, *C. tropicalis*, *S. typhi*, and *C. albicans* using agar well diffusion assay. As per their findings, the essential oil had a strong bactericidal effect; *S. epidermidis* was perhaps the maximum resistance to the concentrated abstraction 29 mm inhibition zones and 0.77 mg/mL MBC), while *C. albicans* was the least (7–9 mm and 24.50 mg/mL MBC) [4]. The literature also reflected that the essential oils of *S. strigillosa* had more antimicrobial activity against Gram-positive bacteria and fungus than Gram-negative bacteria and fungus, as stated by Shen et al. [4]. Pant et al. [90] discovered the antibacterial activity of *S. grossa* essential oils against *K. pneumonia, E. faecalis, B. subtilis,* and *S. enterica* [4]. Skaltsa et al. [91] confirmed that essential oils obtained in Greece from *S. rupestris* and *S. sieberi* had rational antibacterial activity compared to *Staphylococcus aureus* and *B. cereus* [4]. Gousiadou et al. [23] discovered that after exposure to high rates of linalool and nerolidol, the essential oil of *S. albida subsp albida* was highly active against *S. aureus*, *E. coli*, *P. aeruginosa*, *B. subtilis,* and *S. cerevisiae* [4]. Dereboylu et al. [92] examined *Scutellaria* plant species and observed with significant resistance against numerous human pathogenic microbes [93]). Yi Nan et al. [94] discussed total flavones and antimicrobial activity in *S. baicalensis*. Yu et al. [95] discussed Chinese herbal medicine additives in aquaculture. Leung et al. investigated the antibacterial effects of nanoparticles synthesized from *S. baicalensis* [96]. According to the spectrum effect relationships between ultra-performance chromatography and *E.coli B,* incubation with *S. baicalensis* presented significant resistance against *E.coli,* as evaluated by Leach et al. [97].

### 6.2. Enzyme Inhibitory Potential

Acetylcholinesterase (AChE) inhibitors have recently been shown to be an effective clinical strategy for preserving acetylcholine levels and improving cholinergic activity, as stated by Colovic et al. [98]. Inhibiting AChE and Butyrylcholinesterase (BChE) has developed into a standard method for treating the symptoms of neurodegenerative diseases like Alzheimer’s disease [99]. A-amylase and a-glucosidase are two other key glycaemic control enzymes (Shah et al. [1]). The finding of new and reverse tyrosinase enzyme inhibitors, on the other hand, has enabled scientists to develop more accurate Parkinson’s disease prevention measures. The tyrosine-to-dopaquinone conversion catalyzed by tyrosinase may cause neurotoxicity, which has been connected to Parkinson’s disease [100]. Apart from antioxidant activity, aqueous concentrated abstractions of both plants had the lowest inhibitory activity for AChE, a-amylase, and tyrosinase; however, aqueous concentrated abstractions had the highest a-glucosidase inhibition (2.95 and 2.78 mmol/g concentrated abstraction for *S. orientalis* and *S. salviifolia*, respectively). The aqueous concentrated abstractions of both plants did not affect BChE inhibition. Concentrated abstractions of *S. orientalis* and *S. salviifolia*, on the other hand, inhibited AChE (1.37 and 1.69 mg GALAE/g concentrated abstraction, respectively), BChE (1.76 and 1.67 mg GALAE/g concentrated abstraction, respectively), and a-amylase (0.50 and 0.65 mmol ACAE/g concentrated abstraction, respectively) [101,102].

### 6.3. Anti-Fungal Significance

The increase in the complications caused by the fungus and resistance to the marketed available drugs also leads scientists and herbalists to find out new and effective ways to overcome the fungal complications [103]. Previous research has shown that some species of *Scutellaria* and their bioactive ingredients can resist fungal growth [15,104]. The n-Hexane extracted crude oils also have the capacity to resist fungal growth as stated in the literature of Shah et al. [5]. *Scutellaria* also contain bioactive groups such as alkaloid, which is used to break down peptidoglycan stability and degrade fungal cell walls. Another antifungal active compound found in natural herb plants is triterpenoid saponins [105,106]. According to Katzung et al. [107], topical antifungal agents and oral antifungal agents from the azole class can be used to treat candidiasis. According to Ghannoum and Rice [108], the azole antifungal inhibits 14-lanosterol demethylase in the ergosterol synthesis pathway. According to Lyon, Karatela, and Sunay, fluconazole has been identified as an antifungal agent that is effective against the majority of Candida isolates in 2010. Many researchers have recently investigated the case to investigate traditional medicine [109,110].

### 6.4. Anticancer Implication of Genus Scutellaria

*Scutellaria* has anti-metastatic, anti-proliferative, anti-invasion, anti-angiogenic, and apoptosis effects in vitro as well as in vivo [111]. The major constituents of *Scutellaria baicalensis* are wogonin, baicalein, and baicalin [112]. These phytochemicals are not only cytostatic but also cytotoxic to various human tumor cell lines in vitro and inhibit tumor growth in vivo. Most importantly, they show almost no or minor toxicity to normal epithelial and normal peripheral blood and myeloid cells [113]. The antitumor functions of these flavones are largely due to their abilities to scavenge oxidative radicals, attenuate NF-κB activity, inhibit several genes important for regulation of the cell cycle, suppress COX-2 gene expression, and prevent viral infections. The tumor-selectivity of Wogonin has recently been demonstrated to be due to its ability to differentially modulate the oxidation-reduction status of malignant vs. normal lymphocytic cells and to preferentially induce phospholipase Cγ1, a key enzyme involved in Ca_2_+ signaling, through H_2_O_2_ signaling in malignant lymphocytes. Numerous studies have also shown that Wogonin achieves its anticancer effects by modulating a variety of molecular pathways. The major molecular pathways by which it exerts its antitumor effects are reactive oxygen species (ROS), calcium, NF-B, tumor necrosis factor-related apoptosis-inducing ligand (TRAIL), and tumor necrosis factor-alpha, [114] all of which participate in the both intrinsic mitochondria-mediated and extrinsic receptor-mediated pathways [115]. Apart from activating Bax/Bak protein and caspase-8/caspase-9/caspase-3, Wogonin plays a critical function in inhibiting tumor angiogenesis produced by lipopolysaccharide (LPS) or hydrogen peroxide (H_2_O_2_) through the PI3K/AKT/NF-B pathway. Yin et al. examined wogonin to determine its anticancer impact on breast cancer cells. They found that Wogonin might reduce AKT signaling, limit tumor angiogenesis, and finally inhibit tumor development [116]. The toxicity profile of *Scutellaria baicalensis* is given in Table 2.

He et al. [117] stated that wogonin can stop the cell cycle in HCT116 cells in the G1 phase in depends on its dosage by inhibiting the Wnt/β-catenin signaling pathway. Baicalein (5,6,7-trihydroxyflavone) is a flavonoid chemical ingredient mainly isolated from *S. baicalensis* roots. Baicalein is widely used as an anti-inflammatory and anti-cancer agent in Korean and Chinese herbal medicine (Lee et al. [118]). Kim et al. [119] have researched baicalein’s anticancer activity on HCT116 human colon cancer cells and its tumor-preventive potential in mice with colitis-associated cancer. They used azoxymethane (AOM) and dextran sulphate sodium (DSS) to develop colon cancers in mice and examined the impact of baicalein on tumor formation. Baicalein treatment of HCT116 cells inhibited cell growth and induced apoptotic cell death in a concentration-dependent manner. Apoptosis was determined by morphological alterations and poly (ADP-ribose) polymerase cleavage. Baicalein also inhibited NF-kB activation through PPAR-γ activation. These findings suggest that baicalein’s anti-inflammatory actions may be mediated through PPAR-γ activation. Finally, baicalein treatment dramatically reduced the frequency of tumor growth associated with inflammation and data show that baicalein may be a possibility for preventing colon carcinogenesis linked with inflammation.

### 6.5. Anti-Inflammatory Potential

Inflammation is a complication that leads to numerous other pathological disorders. Some *Scutellaria* species are used as a local remedy to treat inflammation. The dominant compounds such as scutellarin, baicalin, alkaloids, saponins, tannins, and glycosides have considerable in vivo pharmacological capacities to cure inflammation, relieve pain, and scavenge the free radicals (Liu et al. [120]). *S. baicalensis* has been found to be a prominent factor to release of oxidative stress and cure inflammation, as described in the literature stated by Huang et al. [121]. *S. edelbergii* has been used for a long time to treat inflammation, which is further validated by the investigation of the screening of *S. edelbergii* in crude extract and sub-fractions and n-hexane extracted crude oils, which possess significant potential to overcome inflammation, as stated in the literature of Shah et al. [5,10]. The EtOAc fraction was the most effective presented (54%) inhibition in comparison to the other examined fractions. They employed Diclofenac sodium as a control, which inhibited inflammation induced by carrageenan by 74% in the experimental mice.

**Figure 5:**
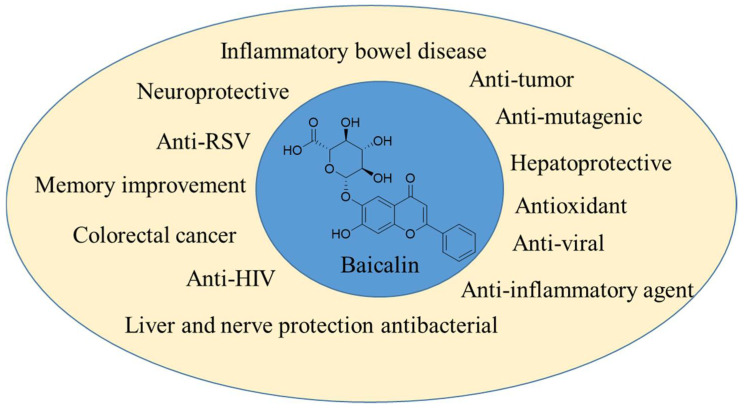
Therapeutic applications of baicalin.

Dogan et al. [122] described the therapeutic role of *S. brevibracteata* in the treatment of inflammation. They sought to decipher the underlying molecular pathways behind stomach inflammatory processes using network pharmacology and molecular docking analysis. We performed gene enrichment analysis and target screening. Nitric oxide (NO) and interleukin-6 (IL-6) cytokines were used for experimental validation in LPS-stimulated RAW 264.7 cells. Additionally, antioxidant activity was determined by examining the radical scavenging effects of various radicals. The isolated compounds were associated with a total of 144 targets, 26 of which were associated with chosen inflammatory targets. The HIF1 signaling pathway and the TNF signaling pathway were identified as being implicated in inflammation by the gene enrichment analysis. Additionally, we designated AKT1, TNF, EGFR, and COX2 as priority targets according to their 26 frequent protein–protein interactions. At 100 and 200 µg/mL, the extract suppressed NO and IL-6 production, respectively, while the flavonoid-rich fraction exhibited considerable anti-inflammatory effects through NO and IL-6 production at 50 and 100 µg/Ml respectively. When combined with the results of network analysis and literature, it is believed that the anti-inflammatory effects of extracts, fractions, and pure compounds were achieved by lowering NO and IL-6 levels via regulation of the NF-B pathway or by lowering NO production via suppression of iNOS via the HIF-1 pathway. The extract and fractions exhibited anti-inflammatory activity that was comparable to that of *S. baicalensis*, a plant widely utilized for its anti-inflammatory properties. Joshee N et al. [123] have highlighted that the genus *Scutellaria* plant species are a prevalent component of Eastern and traditional American medicine. Skullcap is a perennial plant native to North America that is a member of the genus *Scutellaria*. The genus is widespread in the Northern Hemisphere, with almost 400 species. Numerous species are endangered, imperiled, or uncommon. Habitat damage, urbanization, and poor seed set are only a few of the factors contributing to the decline of numerous skullcap populations. Numerous skullcaps feature brilliant, lovely flowers that make them excellent decorative plants. Skullcap is an anti-inflammatory, antispasmodic, emmenagogue, nervine, sedative, and powerful tonic used in alternative medicine. At Fort Valley State University, authors have built a germplasm collection and maintained populations in the greenhouse and by micropropagation. We have achieved great progress in the fields of micropropagation, transformation for desired gene transfer, hairy root induction, and flavonoids, which were observed through HPLC analysis, which is effective for glioma cell lines. According to Mamadalieva NZ et al. [124], the genus *Scutellaria* is represented in Uzbekistan by 32 species, which are used in traditional medicine to treat epilepsy, inflammation, allergies, chorea, and nervous stress. Jia et al. [125] demonstrated that the herbal remedy baicalin induces autophagy in the macrophage cell line Raw 264.7 and results in enhanced Mtb destruction. Additionally, baicalin reduced Mtb-induced activation of the NLRP3 inflammasome and subsequent production of inflammasome-derived IL-1β. To elucidate the molecular processes of baicalin, we studied the signaling pathways involved in autophagy. Baicalin lowered phosphorylated protein kinase B (p-Akt) and phosphorylated mammalian target of rapamycin (p-mTOR) at Ser473 and Ser2448, respectively, but did not affect p38, JNK, or ERK phosphorylation in Raw264.7 or primary peritoneal macrophages. Additionally, baicalin inhibited NF-kB activity. Finally, immunofluorescence experiments revealed that baicalin increased the co-localization of the inflammasome with the autophagosome, suggesting that this may be the underlying mechanism for the autophagic degradation impact on inflammasome activation. Together, baicalin strongly activates autophagy in Mtb-infected macrophages through the PI3K/Akt/mTOR route rather than the MAPK pathway. Additionally, baicalin suppressed the PI3K/Akt/NF-kB signaling pathway, and both activations of autophagy and inhibition of NF-kB contribute to restricting the NLRP3 inflammasome and consequent generation of the pro-inflammatory cytokine IL-1β. They conclude that baicalin is a promising antimycobacterial and anti-inflammatory agent that may serve as a fresh option for the development of new adjunct medications targeting HDT for potential therapy improvement. Therapeutic applications of baicalin are shown in Figure 5.

*Scutellaria galericulata* has been used to isolate two novel flavanone glycosides, dubbed Scugalerosides A and B (1-2). Their chemical structures, including their exact configurations, were determined by an in-depth analysis of physical data. Two novel compounds demonstrated anti-inflammatory activity in vitro, inhibiting the release of -glucuronidase from polymorphonuclear leukocytes of rats by 43.7 and 45.1 percent, respectively, at 10 μM as stated by Xiao et al. [126]

Han QT et al. [127] have isolated two new flavanone glucuronate esters, named Scumoniliosides A and B along with two known flavonoid glucuronate esters, 5,4′-dihydroxyflavonoid-7-*O*-*β*-d-glycuronate methyl ester, and 5,4′-dihydroxyflavonoid-7-*O*-*β*-d-glycuronate butyl ester, from the ethanolic extract of the whole plant of *S.* *moniliorrhiza*. Additionally, their chemical structures were confirmed using integrated spectroscopic techniques, and in vitro studies revealed that four compounds exhibited anti-inflammatory activity, with inhibition rates of -glucuronidase release from rat polymorphonuclear leukocytes ranging from 43.5 to 48.1% at 10 μM. Four undescribed flavonoid alkaloids, as two pairs of enantiomers, were initially isolated as a racemate from the whole plant of *S. moniliorrhiza*. Utilizing chiral HPLC, four isomers, named Scumonilines A-D, were successfully separated, and their chemical structures, including absolute configurations, were established by mass as well as NMR spectroscopy and CD technique. In vitro, four flavonoid alkaloids showed anti-inflammatory activities, with IC_50_ values against the release of β-glucuronidase from polymorphonuclear leukocytes of rats being in the range 5.16–5.85 μΜ. Moreover, four compounds were evaluated for their inhibitory activities against aldose reductase and gave IC_50_ values in the range 2.29–3.03 μΜ. Previously uncharacterized flavonoid alkaloids were first extracted as a racemate from the full plant of *Scutellaria* moniliorrhiza. Four isomers of Scumonilines A-D were effectively separated using chiral HPLC, and their chemical structures, including their absolute configurations, were established using mass, NMR, and CD spectroscopy. Four flavonoid alkaloids demonstrated anti-inflammatory activity in vitro, with IC_50_ values of 5.16–5.8 μM. against the release of β-glucuronidase from polymorphonuclear leukocytes of rats. Additionally, four drugs were tested for their inhibitory activity against aldose reductase, with IC_50_ values ranging between 2.29 and 3.03 μM [128].

### 6.6. Analgesic Significance

**Figure 6:**
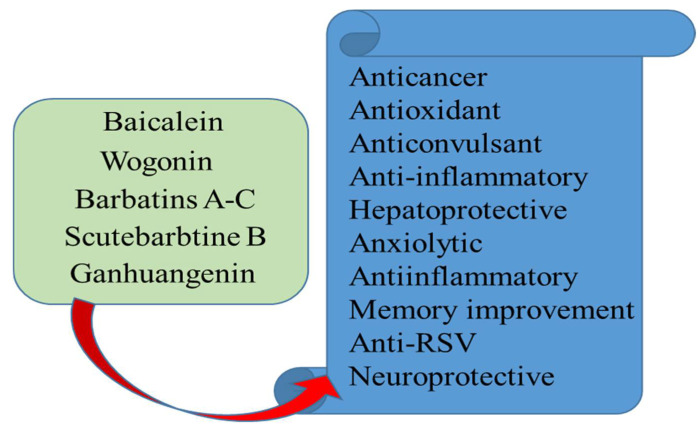
Medicinal uses of the major compounds isolated from *Scutellaria* species.

Some species of the genus *Scutellaria* have the appreciable capacity to assuage pain locally which was further authenticated by the study of Shah et al. [5,10] using various fractions and n-Hexane extracted oils of *S. edelbergii.* The screening presented by [129] also reflected that the species of the selected genus can allay pain. The Baicalin from the roots of *Scutellaria baicalensis* (*S. baicalensis*) and (+)-catechin from the heartwoods of *Acacia catechu* (*A. catechu*) have been used in both over-the-counter joint care dietary supplements and a portion of prescription medical food. Baicalin and catechin anti-inflammatory activity has been reported [130], but there is less work on their analgesic effect. Therefore, author Yimam et al. [131] have used three regularly used animal models of pain to assess the analgesic effect of UP446, a standardized bioflavonoid composition including baicalin and catechins. The antinociceptive efficacy of UP446 oral dosages of (150 mg/kg and 100 mg/kg body weight) was evaluated using carrageenan-induced paw edema, the formalin test, and abdominal constriction tests. Each test employed ibuprofen as a reference chemical. Pretreatment with UP446 at a 150 mg/kg oral dose decreased hypersensitivity to pain by 39.5% in carrageenan-induced hyperalgesia mice. Similarly, a single oral administration of UP446 at a concentration of 100 mg/kg inhibited pain sensitivity by 58% and 71.9%, respectively, in writhing and formalin tests. These results imply that UP446’s standardized anti-inflammatory bioflavonoid content might potentially be used to reduce nociception [132]. Various chemical ingredients reported from the genus *Scutellaria* have some important significance as given in Figure 6.

## 7. Clinical Significance

Medicinal plants are widely used for the ailment of different diseases including viral, bacterial, fungal, parasitic, and various cancer types. According to a clinical trial, the anticancer mechanism of some plant species of the genus *Scutellaria* was evaluated by analyzing biologically active flavonoid, root, leaf, and stem extracts. Breast cancer, prostate cancer, and cells of gliomas were used for tumor-specific effects of *Scutellaria* on cell proliferation, phases of the cell cycle, and apoptosis (MTT assay). It was observed that flavonoids and extracts inhibited the proliferation of breast cancer and gliomas without disturbing noncancerous cells. Similarly, it was involved in extrinsic and intrinsic apoptosis and inhibited G1 and G2 phases of the cell cycle [133]. Recently, an in vitro study reported the effects of *Scutellaria* on cancer cell lines *(A375).* They found that *Scutellaria* inhibited cell proliferation and also suppressed tumor cells [134]. Correspondingly, the authors also suggested that the species included in the genus *Scutellaria* could be used to treat various diseases such as cancer, hepatic disease(s), cardiovascular, and neurogenerative disease. Some pharmacological studies also confirmed the ethnomedicinal significance of the same genus in different disorders including, anticancer, anti-inflammatory, neuro, and hepatoprotective effects [76]. In a recent clinical investigation, due to the extensive promising capacities of the *Scutellaria*, various prescriptions, fractions, and bioactive ingredients isolated from the genus *Scutellaria* have been examined in clinical tests in patients. Radix *Scutellaria* and *S. barbata*, are utilized as the primary modules in combination with other Chinese medicinal aromatic plants in various treatments in China. In the most recent description of the Chinese Pharmacopoeia (2015), with approximately 180 medicines comprising Radix *Scutellariae* registered: *Huangqin Tang* and *Huangqin Gegen Tang* [76].

## 8. Current and Future Perspective

It is estimated that approximately 10% of the 32,000 identified species of plants are medicinally important across the world in which the remedial practices of the genus *Scutellaria* cannot be denied [135]. Because chemically synthesized pharmaceutical products are expensive and out of reach for the average person in developing countries, plants are the primary source of phytomedicines for pain, inflammation, and microbial resistance [136]. Medicinal plants play an important role in clinical research, quality control, disease mitigation, and prevention. The availability of herbal medication is increasing all the time, and it can be managed by screening them for in vitro biological and in vivo pharmacological properties. Due to the high cost of allopathic medicines, ethnomedicinal plant research is essential in developing countries. As a result, determining the relevance of plants and improving plant medication data through novel approaches is critical because they are less toxic and more productive for humanity. Human beings suffering from numerous health complications face drug resistance issues. Microbes have advanced resistance to available antimicrobial drugs, and it has been determined that existing drugs will be completely ineffective against them within the next 30 years [137].

The findings reflected that there is a need to explore more medicinal plants for new therapeutic agents because plant-derived therapy has fewer side effects than drugs prepared through synthesis methods. Another prevalent health issue these days is oxidative stress. Human beings’ food habits, on the other hand, have altered significantly over the last few decades, and our reliance on synthetic materials in foods has grown, leading to an increase in the production of free radicals in our bodies. Oxidative damage is the root cause of many chronic diseases in humans, including inflammation, atherosclerosis, cancer, pain, aging, diabetes, and other degenerative complications [138]. Plant-derived products (fruits, vegetables, herbal medicines) contain a variety of phenolic compounds that, due to aromatic rings, can neutralize free radicals produced within the body and in vitro. Natural antioxidants lower the risk of diabetes, cardiovascular disease, cancer, dermatological infections, and other acute or chronic infections. Even though the bioactivities of a few main compounds (baicalin, baicalein, wogonoside, and wogonin) have been thoroughly researched, several of the chemical compounds personally liable for these actions remain unclear. In recent decades, there is a lot of research into the relationship between *Scutellaria* pharmacological properties and conventional use. Cancer, HIV-1, hepatitis, pain, and pyogenic inflammation are all treated with extracts and specific compounds.

There are a few explanations for this, as per the data reviewed by the researchers, which include: (1) A few of these species were used as ethnomedicine for thousands of years around the world, and particularly Asia and Oceania, and their impacts and stability have been defined. People are becoming more engaged in these plants. In addition, therapeutic approaches may be better to access in some species. (2) Phenols and terpenes have indeed been attributed to two main active compounds of *Scutellaria*. Baicalin, barbatin A–C, baicalein, and scutebarbatine B have all been identified as possible cancer and HIV pharmacogenomics. These compounds will set the foundation for additional investigation on this genus, and they have great potential as novel therapies. (3) However, approximately 35 of the 350 species have been thoroughly investigated. Due to the general abundance of bioactive chemicals across the genus, it is critical to develop different possibilities and develop possible actions. (4) These species have been used in TCM for hundreds of years in China, and the roots of these species are combined with the roots of many other Chinese plant species in a compact design. (5) The essential oils, fatty acid ester, and crude oils of the genus *Scutellaria* also promise therapeutic significance; however, the area that needs to be explored further is isolating the active biopotent chemical constituents. These require documentation as many plant species still lack scientific literature. Our study thoroughly examines traditional uses, botany, pharmacokinetics, phytochemistry, pharmacology, and toxicity, as well as various research proposals. We hope that our findings highlight the importance of *Scutellaria* and encourage its wider distribution.

## 9. Conclusions

Plants have gained serious consideration due to their valuable contribution to nutritional benefits along with health-promoting supplements. These qualities of medicinal plants are due to the presence of vitamins, minerals, and various phenolic ingredients. The genus *Scutellaria* also offered flavonoids, steroids, alkaloids, and tannins. In addition, various compounds such as serotonin and melatonin, as well as baicalin, baicalein, wogonin, scutellarin, and wogonin, were also isolated. In this review article, we have put forward the in vitro and the in vivo studies which include anti-microbial, anti-feeders, anti-malarial, antibiotics, anti-cancer, anti-convulsant, and antioxidant properties. These details may help researchers who are working on isolation and characterization of natural products, medicinal and herbal plants and specifically on the genus *Scutellaria*. *Scutellaria* has numerous pharmacological properties, along with nervous system effects, liver protection, antitumor effects, immune system effects, antibacterial and antiviral impacts, and antioxidant properties. These therapeutic properties imply that *Scutellaria* has a wide range of applications in the treatment of diabetes, depression, and, most importantly, cancer. Because of the presence of triterpenoids and flavonoids, the selected plant could be employed as an antibacterial, analgesic, anti-inflammatory, and antioxidant agent, according to our findings. However, more research is needed to identify the chemicals that are responsible for the observed biological and pharmacological effects.

## Figures and Tables

**Table 1:** Major bioactive compounds identified in the genus *Scutellaria*.

Compounds	Classification	Species	Biological Activities
Aurantiamide acetate	Alkaloid	*S. rivularis* [21]	Anti-tumor, anti-stress, hypo-glycemic [22]
Acetoside	Glycoside	*S. albida* [23]	Antioxidant, cytotoxic [24]
Ajugapitin	Terpenoid	*S. lateriflora* [25]	Antiproliferative [26]
Barbatin A	Flavonoid	*S. barbata* [27]	Antimicrobial [28]
Dihydrocatalpol	Terpenoid	*S. albida* [29]	Antioxidant, anti-inflammatory [30]
Galloyl-O-glucose	Flavonoid	*S. patonii* [31]	Anticancer, antidiabetic [32]
Gallocatechin	Flavonoid	*S. tomentosa* [31]	Anti-inflammatory, anti-diabetic, antioxidant [33]
Gardoside	Glycoside	*S. albida* [23]	Antioxidant, antifungal, anti-inflammatory [34]
Kaempferol-3,7-di-O-rhamnoside	Flavonoid	*S. condensata* [31]	Antiproliferative, antiangiogenic [35]
Lupulin B	Terpenoid	*S. linearis* [36]	Antimicrobial [37]
Leucosceptoside A	Phenol	*S. baicalensis* [38]	Antimicrobial [39]
Lupulin A	Terpenoid	*S. linearis* [36]	Antioxidant, anti-inflammatory, anticancer [40]
Leucosceptoside A	Phenol	*S. baicalensis* [41]	Antioxidant, antibacterial [39]
Myricetin-3’-methyl Ether	Flavonoid	*S. patonii* [31]	Anti-inflammatory, anticarcinogenic [42]
Macfadienoside	Glycoside	*S. albida* [23]	Anti-inflammatory [43]
Melatonin (N-acetyl-5-methoxytryptamine)	-------	*S. leteriflora* [44]	Circadian rhythm dysfunction activity [45]
Pinocembrin	Flavonoid	*S. altissima* [46]	Antimicrobial, anti-inflammatory, antioxidant, anticancer [47]
Procyanidin B1	Flavonoid	*S. pinnatifida* [31]	Antioxidant, antibacterial [8]
Quercetin-3-O-rutinoside	Flavonoid	*S. patonii* [31]	Anti-inflammatory, antimicrobial, antioxidant [48]
Scutellaprostin F	Flavonoid	*S. prostrata* [49]	Antioxidant, antimicrobial [50]
Scutellone D	Terpenoid	*S. rivularis* [51]	Anticancer, anti-inflammation [52]
Scutalpin C	Terpenoid	*S. alpina* [53]	Antibacterial, antiviral [54]
Scutellarein-7-O- neohesperidoside	Flavonoid	*S. multicaulis* [31]	Antioxidant, antimicrobial [55]
Scuteamoenin	Flavonoid	*S. amoena* [56]	Antioxidant, anti-inflammatory [57]
Skullcap flavone I	Flavonoid	*S.baicalensis* [58]	Antioxidant, anti-microbial, anti-inflammatory [9]
Scuteamoenoside	Flavonoid	*S. amoena* [56]	Anti-inflammatory [59]
Saponarin	Flavonoid	*S. condensata* [31]	Anticonvulsant [60]
Scutellaprostin A	Flavonoid	*S. prostrata* [61]	Antiinflammation, anti-cancer [62]
Scutebarbatine B	Terpenoid	*S. barbata* [63]	Antioxidant, antimicrobial, cytotoxic [27]
Scutecolumnin A	Terpenoid	*S. albida* [29]	Anti-inflammatory [64]
Scuteamoenin	Flavonoid	*S. amoena* [56]	Antioxidant, anti-inflammatory [57]
Scutecyprol B	Terpenoid	*S. rubicunda* [65]	Anti-proliferative [66]
Scutalpin M	Terpenoid	*S. alpina* [67]	Antimicrobial [68]
Tenaxin-I	Flavonoid	*S. baicalensis* [69]	Antimicrobial, anti-inflammatory [70]
Tenaxin-I	Flavonoid	*S. baicalensis* [69]	Anti-inflammatory, anti-bacterial [71]
Wogonin	Flavonoid	*S. linearis* [36]	Anti-inflammatory, antioxidant [72]
6-Hydroxy-4-stigmasten -3-one	Steroid	*S. strigillosa* [54]	Antibacterial, anticancer [73]
5,7,2,6-Tetrahydroxy flavone	Flavonoid	*S. baicalensis* [74]	Antimicrobial, anti-angiogenic [75]

**Table 2:** Toxicity and side effects of *Scutellaria baicalensis*.

Compound/Extraction	Cell Lines/Animal	Dose/MTC
EESB	Mice	LD_50_ = 39.60 g/kg
Baicalin	Embryonic stem cell	IC_50_ = 135.9 mg/L
EESB	Rat	2500 mg/kg
Wogonin	Mice	LD_50_ = 286.15 mg/kg

Abbreviations—MTC: minimal toxic concentration, EESB: ethanol extracts of *Scutellaria baicalensis*.

## Data Availability

Not applicable.
